# Liver Diseases and Long Non-Coding RNAs: New Insight and Perspective

**DOI:** 10.3389/fmed.2014.00035

**Published:** 2014-10-17

**Authors:** Luca Quagliata, Luigi M. Terracciano

**Affiliations:** ^1^Molecular Pathology Division, Institute of Pathology, University Hospital of Basel, Basel, Switzerland

**Keywords:** lncRNAs, liver diseases, hepatocellular carcinoma

## Abstract

The new landscape of human transcriptome along with the identification of numerous long non-coding RNAs (lncRNAs) has dramatically altered our approach to study diseases. It is now imperative to decipher the biological functions of these transcripts and how they impact on human cell and pathophysiology. Nonetheless, already at this very early stage of their study, the involvement of lncRNAs in cell transformation is emerging as a key aspect. Recently, researchers have started to explore the implications of lncRNAs alteration in hepatic pathophysiology. In this review, we will discuss in detail several examples of liver disease-relevant lncRNAs. Many lncRNAs have been shown to play a major role in hepatocellular carcinoma (HCC). For such type of tumor with an increasing incidence and a high mortality rate, it is crucial to identify new therapeutic targets and biomarkers to predict response to therapy. LncRNAs present as a promising new resource. One major challenge for the future would be to systematically address the lncRNAs expression among the different cellular components of the liver. To achieve this goal, a combination of clinically driven, genetically defined, morphologically classified, and molecular-based studies will have to be performed. In conclusion, lncRNAs will undoubtedly provide a rewarding field of study and most importantly a new resource to identify new disease associated biomarkers and molecular targets for therapy for liver diseases.

## Introduction

The ENCODE (Encyclopedia of DNA Elements) project has substantially contributed to reveal that more than 70% of the human genome is transcribed (1), but only as few as the 2% of produced RNA is ultimately translated into proteins (2). The discovery of numerous non-coding RNA (ncRNA) transcripts in humans has dramatically altered our understanding of complex diseases such as cancer. The scientific community now faces the challenge to unravel the biological functions of these transcripts and to understand how they impact on human cell physiology (2). In general, ncRNAs are grouped into two major classes based on their length. Those transcripts shorter of 200 nucleotides (nt) belong to the class of small ncRNAs, mainly including Piwi-interacting RNAs, small interfering RNAs, and microRNAs (miRNAs). Long non-coding RNAs (lncRNAs) are mRNA-like transcripts ranging in length from 200 nt to circa 100 kb, yet are poorly conserved and do not function as templates for protein synthesis. However, more recently, among ncRNAs, the landscape of lncRNAs has been unveiled by the fast progress of deep sequencing technology along with the development of bioinformatics tools enabling their identification (3). Based on their genomic proximity to protein-coding genes, lncRNAs have been classified into four classes: exonic lncRNAs, intronic lncRNAs, overlapping lncRNAs, and intergenic lncRNAs. However, the definition of lncRNA, far for being comprehensive, is still under debate and rapidly changing as researchers collect more information about them (4). Up to date, lncRNAs are described as mRNA-like 5′-capped, polyadenylated RNAs transcribed by the RNA polymerase II (RNA pol II) but with no evident open reading frame (ORF), mostly acting to organize and direct ribonucleoprotein (RNP) complexes, thus controlling gene expression (2, 4). LncRNAs are proposed to be the largest transcripts class in the mouse and human transcriptomes. Several lncRNAs have been shown to play essential roles in biological events such as embryogenesis and tumorigenesis by regulating fundamental cellular processes such as stress response, proliferation, differentiation, and survival. Noteworthy, this class of genes have shown to be poorly conserved across the species (5), and, differently from coding genes, their number seems to be directly related to the level of organism complexity (2). Interestingly, according to the ENCODE project data, the number of postulated lncRNAs should be higher than protein-coding genes (4–6). One major characteristic of lncRNAs is their ability to fold into complex secondary structures, something that is not feasible for short sequence RNAs such as miRNAs, thus for example, enabling lncRNAs to provide docking station for protein complexes aggregation (5). Although for the majority of lncRNAs all potential functions and the mechanisms of action are still undetermined, one major finding is that most lncRNAs show a tissue-specific pattern of expression and are as well differentially abundant across several stages of development (6). lncRNAs transcription indeed occurs in a tidily regulated manner restricted both in time and space constraints (6). Among the many functions that have been ascribed to lncRNAs, the following are particularly noteworthy: decoy activity to bind and titrate away proteins (7–9); guiding function to direct the localization of protein complexes to specific DNA region (10, 11), either *in cis*, thus close by their genomic locus, as for example HOTTIP (HOXA transcript at the distal tip) (11), or *in trans*, controlling genomic distant chromosomal region, e.g., HOTAIR (HOX Transcript Antisense RNA) (12); acting as scaffold or adaptors providing a platform for large molecular complexes assembly (13). In addition, sponge-acting lncRNAs, able to sequester by binding and thus eventually inhibiting miRNA activity (14, 15), have gained a lot of attention in the field (16). However, whether this is a physiologically relevant phenomenon or not is still under debate (16, 17). Recently, a bunch of manuscripts have started to explore the implications of lncRNAs alteration in hepatic pathophysiology (18). We will discuss in detail several examples of liver disease relevant lncRNAs in the next paragraphs. Since these genes do not code for proteins, the role of their transcripts is challenging to be studied when functional experiments are hard to be performed. For example, classical loss of function approach using siRNA has been shown to be tricky to be accomplished (19). At the same time, considering that many lncRNA loci overlap with other genes, targeted gene recombination aiming to disrupt gene expression has also shown to be problematic (19), thus resulting in hard to interpret phenotypes. Exploring lncRNAs functions will be critical to fully appreciate the relevance of these genes in the biological processes controlling liver functionality. Nonetheless, already at this very early stage of their study, the involvement of lncRNAs in cell transformation is emerging as a key aspect (1).

## lncRNAs in Hepatocellular Carcinoma

Hepatocellular carcinoma (HCC) is the third leading cause of cancer-related death worldwide and represents the majority of liver cancers (80% circa of all cases) (20, 21). HCC mostly arises in cirrhotic liver and is indeed the principal cause of mortality among cirrhotic patients (21). In addition, HCC has been proven to be highly refractory to treatment (22). Unlike most malignancies, mortality from liver cancer has increased significantly over the past 20 years (23). Around 70% of new HCC cases worldwide (with higher prevalence in Eastern countries) are related to chronic infection with either Hepatitis B (HBV) or C (HCV) virus (24). Epidemiological evidence concerning the prevalence of chronic hepatitis affected patients indicates that the medical and economic burden of liver cancer will still increase significantly in Western populations during the next decades (23, 25). Besides well-known risk factors, such as male gender, older age, viral infection and genotypes, alcohol intake, diabetes, obesity, increased portal hypertension, and ethnicity (20, 24, 26), little is known about the mechanisms that favor HCC development and progression (20).

From a molecular perspective, HCC is a highly heterogeneous tumor reflecting under this aspect the wide range of its etiologic-associated factors (24, 27). Unbiased molecular approaches such as global gene expression profile (either microarray based or massive RNA sequencing) along with DNA deep analysis (next generation sequencing, NGS) have identified key alterations in HCC involving regulatory pathways such as Wnt/β-catenin, loss of Axin1 and Axin2, MAPK, p14ARF/p53, p16INK4A/Rb, transforming growth factor-β (TGF*-*β), and PTEN/Akt (26–29). However, although substantial efforts have been placed in the identification of important molecular players in hepatocarcinogenesis, currently, all HCC cases presenting with the same stage are treated irrespectively of their molecular subtypes. Thus, the identification of new HCC makers is at the same time an unmet need and a great opportunity to establish more personalized therapies. One major contribution aiming to ameliorate the current molecular classification of HCC may occur by the integration of lncRNAs expression profiles into existing data sets. Up to date, a continuous growing number of lncRNAs are described to be involved in HCC disease development and progression (1, 3, 30). In Table 1 some of the best-characterized lncRNAs involved in HCC are shown. For example, HULC (highly upregulated in liver cancer) expression among the first lncRNA to be investigated in HCC is increased in HCC samples being as well associated with histological grade and HBV infection (31). HULC oncogenic activity in HCC has been partially uncovered via gain and loss of function experiments demonstrating that it promotes proliferation of hepatoma cells through suppressing p18 (32). Furthermore, increased HULC levels have been also detected in the plasma of HCC patients (33). Importantly, these findings suggest that lncRNAs in plasma might be used as non-invasive novel biomarkers for the diagnosis and/or prognosis as well as monitoring of disease progression. H19 levels, an lncRNA that is exclusively expressed by the maternal allele, are associated with IGF2 (insulin-like growth factor 2) and are increased in HCC; high H19 levels are associated to worst patients’ prognosis (34, 35). MALAT-1 (metastasis associated lung adenocarcinoma transcript 1), with a remarkable RNA transcript length of 8.7 kb, is associated to metastasis formation and HCC recurrence (36). TUC338 (transcribed ultra-conserved region 338) levels are also altered in HCC and peri-tumoral cirrhotic tissue (37). Interestingly, TUC338 knockdown reduces tumor growth in both murine and human HCC cell lines (37). Our group has also contributed to this field and has recently discovered that HOTTIP, an HOXA locus associated lncRNA, is highly upregulated in HCC and can predict both disease progression and patients’ outcome (38). HOTTIP is located in physical contiguity (chr 7p15.2) with the transcriptional factor HOXA13 (11). Consistent with its genomic position 5′ to HOXA13, HOTTIP is exclusively expressed from development to adulthood in lumbo-sacral anatomic regions (11). Via interacting with the WDR5/MLL protein complex and guiding it to precise DNA loci, HOTTIP directly coordinates and controls the activation of several 5′ HOXA genes (11). Furthermore, HOTAIR expression, another HOX genes associated lncRNA that resides within the HOXC locus close by HOXC11 and is specifically expressed in distal and posterior anatomical structures, can predict HCC recurrence after liver transplant (39). At the same time, its inhibition *in vitro* reduces cell invasion and increases chemosensitivity (40). HOTAIR interacts with chromatin remodeling complexes, such as PRC2 and LSD1/CoREST-H3K4, to respectively methylate or de-methylate DNA (13, 41). Among the latest example reported in literature, the lncRNA-activated by TGF-(lncRNA-ATB) is upregulated in HCC and associated with poor prognosis (42). lncRNA-ATB upregulates ZEB1 and ZEB2 by competitively binding the miR-200 family members thus inducing EMT and invasion eventually resulting in metastasis in HCC patients. In addition, lncRNA-ATB promotes organ colonization of disseminated tumor cells by binding IL-11 mRNA and triggering STAT3 signaling. These data suggest that lncRNA-ATB may serve as a potential target for anti-metastatic therapies.

**Table 1:** **Examples of IncRNAs associated with liver diseases**.

IncRNA	Transcript (kb)	Reported role in liver disease	Reference
HULC	0.5	High expression in HCC is associated with tumor grade and HBV infection	(31, 32)
H19	2.3	Overexpressed in HCC and peri-tumoral area, correlates with prognosis	(33, 34)
MALAT-1	8.7	Increased in HCC, it is associated with metastasis and disease recurrence	(35)
TUC338	0.59	Controls cell proliferation and is increased in HCC and cirrhosis	(36)
HOTTIP	4.3	Highly upregulated in HCC, predics disease progression and outcome	(11, 37, 40)
HOTAIR	2.3	Associated to cell invasion and chemosensitivity, predicts disease recurrence	(12, 13)
IncRNA-ATB	2.7	Controls EMT via ZEB1/ZEB2, predicts survival	(41)
HELLP	2.4	Linked to a Mendelian disorder with autosomal-recessive inheritance	(43)
lncRNA-LALR-1	1.2	Enhance hepatocyte proliferation capacity after partial hepatectomy	(44)
IncRNA DYNLRB2–2	10.2	Modulate both glucose and cholesterol metabolism in liver	(45)

One major unmet need in HCC is the lacking of clinically validated response to therapy markers. When detected at an early stage of disease, HCC patients might be still eligible for surgical-based curative treatments (20). However, currently only 30–40% of HCC patients are diagnosed at an early stage and are eligible for resection, local ablation, or transplantation (20). Conversely, when HCC is detected at an intermediate or advanced stage, no treatment option exists besides Sorafenib (46). Sorafenib is a multityrosine kinase inhibitor that effectively blocks several receptors activity such as VEGFR (vascular endothelial growth factor receptor), PDGFR (platelet-derived growth factor receptor), and the RAF serine/threonine kinases along the RAF/MEK/ERK pathway (46). Nevertheless, Sorafenib has been shown a consistent but limited survival benefit in HCC (10–12 weeks increased survival) accompanied by a number of moderate to severe side effects (43, 46). This makes HCC somehow unique among cancers having no standard cytotoxic therapy. Thus, it is imperative to identify new therapeutic targets and biomarkers to predict response to therapy. LncRNAs present as a promising new resource.

## lncRNAs in Non-Neoplastic Liver Diseases

Although most of the so far characterized lncRNAs investigated in liver have been shown to influence liver cancer development and/or disease progress (1), several others lncRNA have been also associated to non-neoplastic liver conditions. For example, using genome-wide linkage analysis of families with HELLP syndrome, a disorder defined as a group of symptoms that occur in pregnant women mostly during the third trimester having recurrent hemolysis, low platelet count, and markedly elevated liver enzymes, it has been shown that such condition is associated to a specific haplotype present on the 12q23 chromosome region (47). Further studies have demonstrated that this genomic locus, more precisely in the intergenic region between the PMCH and IGF1 genes, contains an lncRNA transcript more than 205 kb in length (47). So far, this is the first lncRNA gene to be linked to a Mendelian disorder with autosomal-recessive inheritance with direct effect on liver functionality (44, 47). Furthermore, the lncRNA named LALR-1 (human ortholog hLALR-1) has been shown to enhance hepatocyte proliferation capacity both *in vitro* and *in vivo*, eventually promoting liver regeneration capacity after 2/3 partial hepatectomy (PH) experiments. LALR-1 facilitates cyclin D1 expression through the activation of Wnt/β-catenin signaling via the suppression of Axin1 (45). It should be now investigated whether targeting hLALR-1 *in vivo* may be therapeutically beneficial in liver failure condition or liver transplant where sustained liver regeneration is needed. Finally, increased liver levels of the lncRNA DYNLRB2–2 inducing GPR119G (protein-coupled receptor 119) and ABCA1 expression has been shown to modulate both glucose and cholesterol metabolism in liver, eventually resulting in reduced atherosclerotic plaque progression in apoE(−/−) mice (48). These results suggest that the modulation of DYNLRB2–2/GPR119 liver levels might offer a therapeutic approach to prevent plaque formation in high-risk atherosclerotic prone individuals.

## lncRNAs in Liver Diseases: A Glimpse into the Future

Long non-coding RNAs are increasingly recognized as essential regulators of biological processes in normal and pathological conditions thus having a high potential to serve as novel diagnostic and prognostic tools (49). Many individual examples of disease relevant lncRNAs have been reported, but research in the field of liver-associated disease is somehow still left behind. Deep biochemical and functional analyses of cells with specific-lncRNA altered expression are a first step to advance toward a more comprehensive understating of their physiological role. However, one major challenge for the future would be to systematically address the distribution of lncRNAs expression among the different cellular components of the liver. Whether hepatocyte, Kupffer, endothelial, or bile duct cells, it will be critical to identify where and to what level lncRNAs are expressed within the liver. At the same time, defining the localization of specific lncRNAs will also provide additional essential information concerning their functions. Considering the prominent involvement of lncRNAs in HCC development and progression, another challenge for researchers in the field will be to assess the lncRNAs landscape in cirrhotic patients. This approach might eventually lead to the identification of lncRNAs that could be used as predictive biomarkers of disease development. To achieve this goal, a combination of clinically driven, genetically defined, morphologically classified, and molecular-based study will have to be performed. Multidisciplinary research group with differential background, from clinicians to geneticists, pathologists, molecular biologists, and biochemists, should cooperate to successfully address these issues. To conclude, lncRNAs will certainly provide a fruitful field of study and most importantly a new resource for disease associated biomarkers and molecular targets for therapy for liver diseases.

## Conflict of Interest Statement

The authors declare that the research was conducted in the absence of any commercial or financial relationships that could be construed as a potential conflict of interest.

## Abbreviations

ENCODE, encyclopedia of DNA elements; HCC, hepatocellular carcinoma; HBV, hepatitis B virus; HCV, hepatitis C virus; HOTAIR, HOX transcript antisense RNA sequencing; HOTTIP, HOXA transcript at the distal tip; HULC, highly upregulated in liver cancer; IGF2, insulin-like growth factor 2; IHC, immunohistochemistry; kb, kilobase; MALAT-1, metastasis associated lung adenocarcinoma transcript 1; miRNAs, microRNA; ncRNA, non-coding RNA; NGS, next generation sequencing; nt, nucleotides; ORF, open reading frame; PH, partial hepatectomy; RNP, ribonucleoprotein; RNA pol II, RNA polymerase II; RNA-seq, RNA sequencing; TUC338, transcribed ultra-conserved region 338.
